# Machine-Learning-Enabled Virtual Screening for Inhibitors of Lysine-Specific Histone Demethylase 1

**DOI:** 10.3390/molecules26247492

**Published:** 2021-12-10

**Authors:** Jiajun Zhou, Shiying Wu, Boon Giin Lee, Tianwei Chen, Ziqi He, Yukun Lei, Bencan Tang, Jonathan D. Hirst

**Affiliations:** 1 Key Laboratory for Carbonaceous Waste Processing and Process Intensification Research of Zhejiang Province, University of Nottingham Ningbo China, 199 Taikang East Road, Ningbo 315100, China; Jiajun.Zhou@nottingham.edu.cn (J.Z.); Shiying.WU@nottingham.edu.cn (S.W.); shytc1@nottingham.ac.uk (T.C.); shyzh1@nottingham.ac.uk (Z.H.); shyrl2@nottingham.ac.uk (Y.L.); 2 School of Computer Science, University of Nottingham Ningbo China, 199 Taikang East Road, Ningbo 315100, China; boon-giin.lee@nottingham.edu.cn; 3 School of Chemistry, University of Nottingham, University Park, Nottingham NG7 2RD, UK

**Keywords:** LSD1, LSD1 inhibitors, machine learning, virtual screening

## Abstract

A machine learning approach has been applied to virtual screening for lysine specific demethylase 1 (LSD1) inhibitors. LSD1 is an important anti-cancer target. Machine learning models to predict activity were constructed using Morgan molecular fingerprints. The dataset, consisting of 931 molecules with LSD1 inhibition activity, was obtained from the ChEMBL database. An evaluation of several candidate algorithms on the main dataset revealed that the support vector regressor gave the best model, with a coefficient of determination ($R^{2}$) of 0.703. Virtual screening, using this model, identified five predicted potent inhibitors from the ZINC database comprising more than 300,000 molecules. The virtual screening recovered a known inhibitor, RN1, as well as four compounds where activity against LSD1 had not previously been suggested. Thus, we performed a machine-learning-enabled virtual screening of LSD1 inhibitors using only the structural information of the molecules.

## 1. Introduction

Epigenetic mechanisms are fundamental in genome-dependent biological processes. By performing an important role in regulatory effects, epigenetic mechanisms participate in gene expression and transcription coordinated by the DNA sequence [1]. These mechanisms encompass a wide spectrum of biological activities and develop dynamic regulation in gene transcriptional modulation, genome reprogramming modification, and homeostatic maintenance [2]. As one of the key epigenetic processes, histone modification is responsible for part of transcriptional regulation. Lysine-specific histone demethylase 1 (LSD1) is the first histone demethylase discovered to act as a dynamic modulator in genome transcriptions of cellular processes. This regulation is specifically achieved by LSD1 catalysing the oxidative demethylation of mono and dimethylated histone H3 at Lys4 and Lys9 [3]. The methylation of different types of lysine substrates in histone is attributed to both positive and negative regulatory effects. This regulatory mechanism targeting methylated histone H3 exhibits activation on transcription when the substrate is Lys4 and repression when the substrate is Lys9 [4]. Therefore, LSD1 mediates a number of cellular signaling pathways and participates in key modifications of gene expression. The high functional diversity of histone methylation in living cells explains the potential connection between the dysfunction of LSD1 and various pathological conditions, such as viral diseases and neurodegeneration [5]. The aberrant overexpression of LSD1 has been closely linked with the tumorigenesis and progression of several cancers [3,5,6]. Under the inhibitive state of LSD1, suppressive gene expression against cancers can be activated with an increased degree of methylation [7,8]. Some biologically potent compounds can induce the inactivation of LSD1 inhibition, which implies that the chemical suppression of cancer cells from proliferation, migration, and invasion is feasible [9,10].

Recognized as a promising strategy for cancer treatment, as discussed in an earlier review [11], several potential LSD1 inhibitors have been discovered, including GSK-2879552, INCB059872, and RG6016, and have progressed to the stage of clinical trials [12,13,14]. Although current advances facilitate the use of prior knowledge towards the discovery of new LSD1 inhibitors, rational and effective design remains a challenge. Recently, some approaches to rational design have exploited structural similarities in both the LSD1 protein substrate and the known inhibitors. LSD1 is structurally homologous to the members of the monoamine oxidase family: MAO-A and MAO-B. Based on the homology, it was hypothesized that an inhibitor of monoamine oxidase might also suppress LSD1 in a similar manner [15]. The application of protein structure similarity clustering gave similarity scores between the LSD1 and MAOs, which encouraged work to expand the use of γ-pyrones inhibitors from MAOs to LSD1 [16].

There have been several computational studies of inhibitors of LSD1, using techniques such as pharmacophore modelling, 3D-QSAR, and molecular docking. In one study [17], CoMFA [18] was used to generate a 3D-QSAR model of 41 stilbene derivatives, and this was supplemented with molecular docking and molecular dynamics simulations. A similar approach was adopted in a study of some thieno[3,2-b]pyrrole-5-carboxamide derivatives [19] and in a study of some tranylcypromine derivatives [20]. In a study of 29 5-hydroxypyrazole analogues [21], descriptors derived from molecular docking were used in multiple linear regression and support vector machines to generate predictive QSAR models, albeit on a small dataset. 2D- and 3D-QSAR models achieving similar accuracies (and with the same caveat) have also been built for 54 aminothiazole and thiazolesulfonamide derivatives [22].

Molecular docking also provides a computational tool to predict binding affinity and evaluate protein–ligand interactions. Compounds containing a propargylamine warhead were virtually screened from a library inspired by inhibitors of MAOs, and validated by docking analysis [9]. The computational tools also helped to extend the chemical search space to large and diverse compound libraries to realize a high-throughput virtual screening. By establishing a quantitative structure–activity relationship (QSAR), the initial hits discovered by computational docking were optimized to have better drug-like properties [3].

Virtual screening approaches to discover new inhibitors of LSD1 have also been an area of interest. A template virtualization technique combined with standard similarity search techniques was reported [23], which led to the discovery of 27 new validated hits, the best having a potency of 0.2 μM. Another virtual screen based on a pharmacophore model combined with docking identified 9 validated hits, with micromolar potency [24]. Virtual screening with a pharmacophore also suggested that compounds with a 3-methylxanthine scaffold may be a fruitful strategy to pursue [25].

Recently, the rapid development of machine learning has attracted the attention of researchers in computational chemistry and drug discovery [26,27,28,29,30,31]. Machine learning often demands a large quantity of high-quality data to reach a useful level of predictive accuracy [32]. Compared with traditional fields where machine learning is advantageous, chemistry-related fields often suffer from the expensive acquisition of chemical data, which is a bottleneck. In this regard, we turned to publicly available databases to acquire sufficient data to build a reliable model. We developed a machine learning model using a variety of algorithms that have not previously been considered. This model is based on a significantly larger set of molecular structures of LSD1 inhibitors than has previously been considered, and we utilised the model in virtual screening.

## 2. Methods

### 2.1. Data Collection

A dataset containing compounds targeting LSD1 was assembled from the ChEMBL database (version 28) [33], comprising the molecule and the inhibition assay result of each example. The data in ChEMBL, which is open access, are abstracted and curated from primary scientific literature and comprise compound structures and their biological activities, which is our particular focus. The molecules were represented by the Simplified Molecular-Input Line-Entry System (SMILES) [34]. Assay descriptions, along with ChEMBL documents extracted by indexing the document ID, were used to identify and screen out the comparable biological activities that exhibit the features of LSD1 inhibition. The activity measurements were utilized directly in our model and treated as our regression target. The pChEMBL values were used, because this allows (with some caveats) one to use several types of bioactivity measurements, including molar concentrations of $IC_{50}$, $XC_{50}$, $EC_{50}$, $AC_{50}$, $K_{i}$, and $K_{d}$, on a negative logarithmic scale [35], as shown in Equation (1), where the effective value is any of the preceding quantities.

(1) $$pChEMBL=-\log_{10}\left(Effective\;Value\right)$$

Some molecules had multiple measurement results from different assays on the LSD1 inhibition, which might affect the efficiency and performance of machine learning algorithms. Therefore, duplicate structures were removed and the mean value of each measurement was calculated. The mean value was transformed to a pChEMBL value. A total of 931 distinct instances are included in the final dataset.

### 2.2. Molecular Fingerprints

A recent study by Sandfort et al. [36] suggests that the use of structure-based descriptors can lead to a predictive accuracy of activity comparable to that from models built with numerical quantum descriptors. Thus, in our study, we have focused only on structural-based descriptors. To provide structured input data for the machine learning algorithms, the molecules were transformed to one of the molecular fingerprints. The Morgan fingerprints, developed from Morgan algorithms, were chosen due to their wide applicability [37]. Using the RDKit package (version 2020.09.1), each SMILES string was converted to a Morgan bit vector of a predefined length (*L*) comprising a series of binary bits. For circular fingerprints, the radius (*r*) is a key variable, as it encodes the neighbouring environment around the central atom. The radius determines the number of iterations in the calculation of the identifier of the central atom. With the increase of the radius, the information of the surrounding substructure is increasingly encoded into the identifier [38,39]. Each identifier is updated iteratively to include information on neighbouring atoms (i.e., their identifier and bond order). Once the iterations have reached the specified radius, the identifiers are folded into the length of the bit vector using a hashing function. In this manner, Morgan fingerprints were calculated for the main dataset with *L* = 512 and *r* = 3 for evaluation of the performance of machine learning methods and the construction of a model for virtual screening for new LSD1 inhibitors.

### 2.3. Model Construction

Several machine learning algorithms were built and tuned in the scikit-learn package (version 0.22.2) [40]. A multi-layer perceptron (MLP) was also trained using the PyTorch package (version 1.8.1) [41] with CUDA (version 10.1) [42] under the Google Colaboratory environment. The dataset was first randomly divided into a training set and a test set with a split of about 80:20 (744:187). Based on the training set, for each algorithm, a specified pool of hyperparameters was optimized with a five-fold cross validation strategy to find the combination that achieved minimal loss. This strategy maximises the use of limited data in a relatively small dataset. The test set was excluded from the training and validation process as a ‘holdout’ dataset and used only to test each model’s predictive capability on unseen data. The algorithms predict continuous variables, so performance was evaluated by the coefficient of determination score ($R^{2}$) and the root-mean-square error (RMSE). Both metrics were applied on the training set to evaluate the fitting ability and test set to evaluate the generalizability of the machine learning model. The fine-tuned algorithm with the highest $R^{2}$ and the lowest RMSE on the test set was deployed in the virtual screening.

### 2.4. Virtual Screening

Using the developed model, virtual screening was applied to the ZINC 15 in-vitro dataset, which contains 306,347 molecules [43]. Whilst there are many possible libraries that could be screened, we have focused initially on ZINC 15, which is a particularly well established and widely used library containing bioactive and drug-like molecules. Each molecule was represented by Morgan fingerprints. A ‘hit’ in the virtual screen was defined as a molecule with a predicted pChEMBL value of 7 or more.

## 3. Results and Discussion

### 3.1. Characterisation of the Dataset

**Figure 1:**
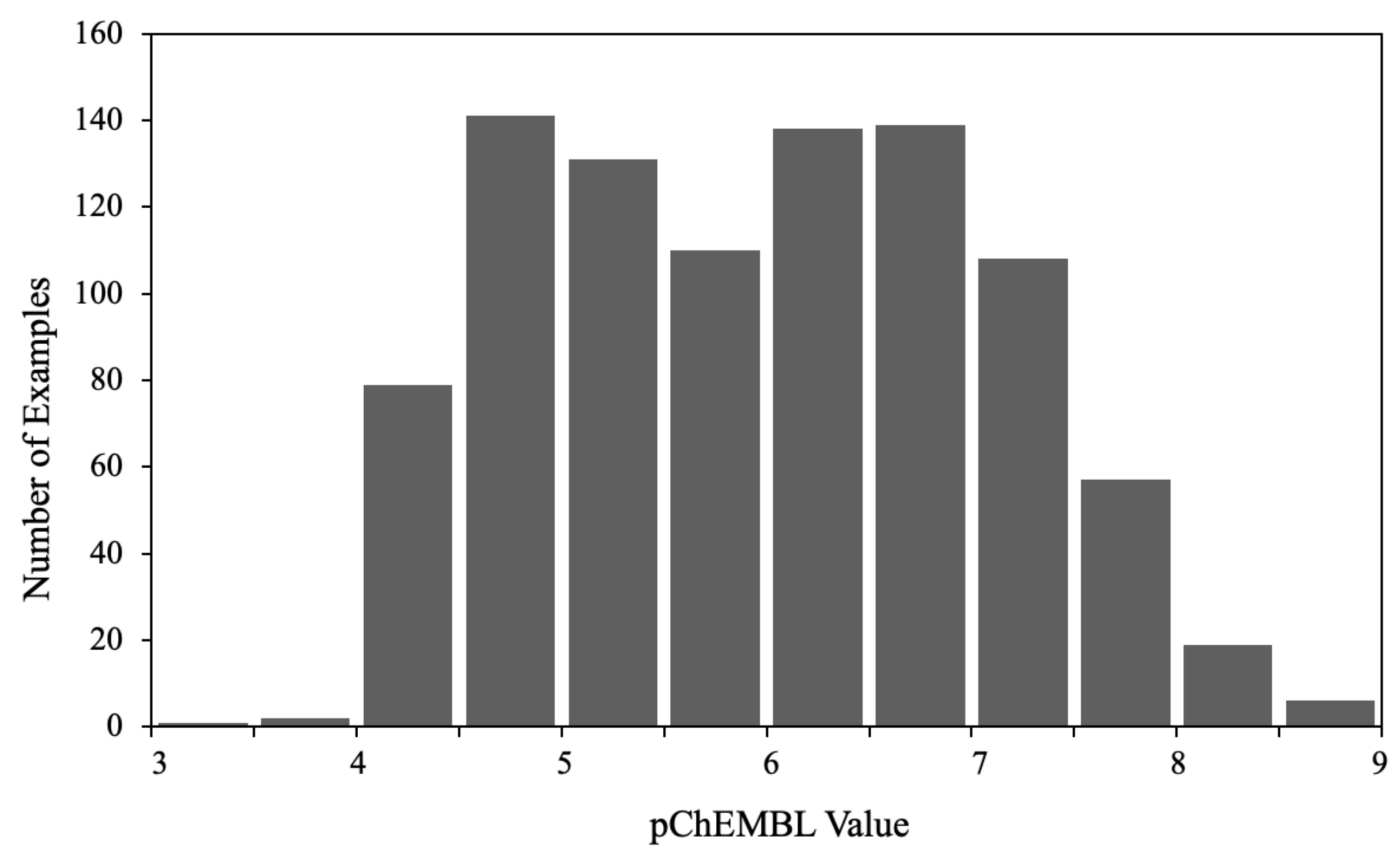
Distribution of the activities of the molecules in the dataset.

The molecules in the dataset cover a considerable range of LSD1 inhibitory activity (Figure 1), which is important for machine learning algorithms to model the quantitative structure–activity relationship well. Based on an effective $IC_{50}$ value of 100 nM, 190 compounds (20.4 %) had an activity above 7, while 741 compounds (79.6 %) were below 7.

**Figure 2:**
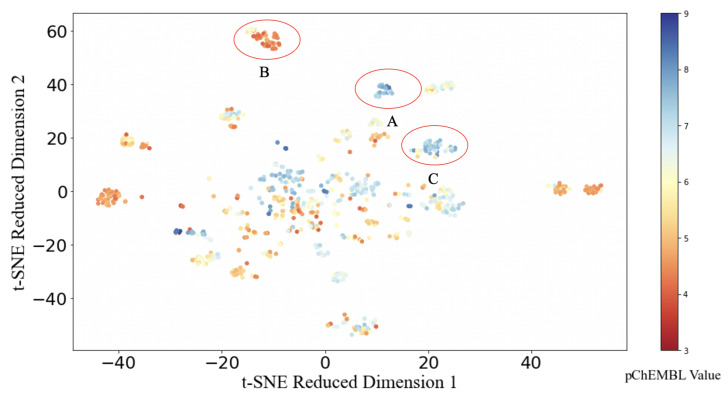
Dimensionality reduction by t-distributed Stochastic Neighbour Embedding (t-SNE) on fingerprint bit vectors. The colourbar indicates pChEMBL values from 3 (red) to 9 (blue).

In order to visualize the mapping between the structural features and LSD1 inhibition in two dimensions, t-distributed Stochastic Neighbour Embedding (t-SNE) was applied, due to its ability to preserve local data structures from original high dimensional space while presenting clustering information. This nonlinear dimensionality reduction technique considers the similarity between the pairs of points in their original high dimensional space and their target two-dimensional embedding. The t-SNE algorithm minimizes the Kullback–Leibler divergence between the vector of similarities between pairs in the original high dimensional space and the pairs embedded in the two-dimensional mapping [44,45]. A short Euclidean distance between pairs of data points in Figure 2 indicates a significant extent of structural similarity. Several clusters are evident in the two-dimensional map.

**Figure 3:**
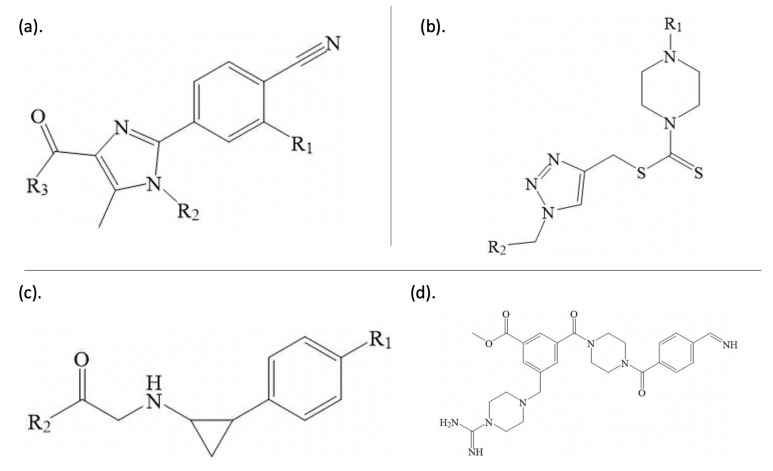
Core structures of molecules in Clusters A, B, and C from the t-SNE analysis, shown in (**a**–**c**), respectively. $R_{1}$ in (**c**) is shown in the *para* position, but there are also compounds in this cluster with $R_{1}$ in the *meta* position. (**d**) shows the full structure of one unusual molecule from Cluster C, which in fact does not have the core structure shown in (**c**).

In Figure 2, A, B, and C are marked out as clusters with a clear separation from other data points. The molecules in Cluster A (Figure 3a) have a common core structure. The molecules in Cluster B share a common core structure that is distinct from that of Cluster A (Figure 3b). However, on the periphery of Cluster C, one molecule (Figure 3d) does not share the core structures of the other molecules in Cluster C. This different molecule also exhibits the lowest activity of the molecules in Cluster C. Several clusters of molecules sharing nearly identical core structures and similar activities (denoted by aggregations of similar colours in Figure 2 are strong evidence of the existence of a quantitative structure-activity relationship.

Some structural features in Figure 3 are noteworthy. Clusters A and C correspond to high activity compounds and are, thus, of particular interest. For the common structure in Cluster A (Figure 3a), the $R_{3}$ substituent is a five- or six-membered ring with a nitrogen atom connected to the common structure. For the common structure in Cluster C (Figure 3c), $R_{1}$ is located at either the *meta* or *para* position of the benzene ring. $R_{2}$ is also a five- or six-membered ring with a nitrogen atom connected to the common structure.

### 3.2. Performance of the Machine Learning Algorithms

Several commonly used machine learning algorithms were applied for comparison and evaluation. The predictive ability of each algorithm, shown in Table 1, was evaluated by the coefficient of determination ($R^{2}$) and the root-mean-square error (RMSE). Mean values of $R^{2}$ and RMSE and their standard deviations were calculated to assess the stability of the algorithms over different train–test splits of the dataset. The support vector regressor (SVR) with the radial basis function (RBF) kernel achieved both the highest $R^{2}$ and the lowest RMSE on the test set among all optimized models. The SVR slightly outperformed the random forest regressor (RF) by 0.8%. As a baseline model, the simple decision tree regressor (DT) was the least predictive, but the test $R^{2}$ of 0.42 suggests that the lower bound for the performance of machine learning models in predicting for this QSAR is actually quite high. In addition, the models that achieved high predictive accuracy in the test set also show excellent performance on the training set. The very low standard deviations of the best two models indicate that the SVR and RF exhibit good stability. Changes to the training set cause only minor fluctuations in the predictive performance.

**Figure 4:**
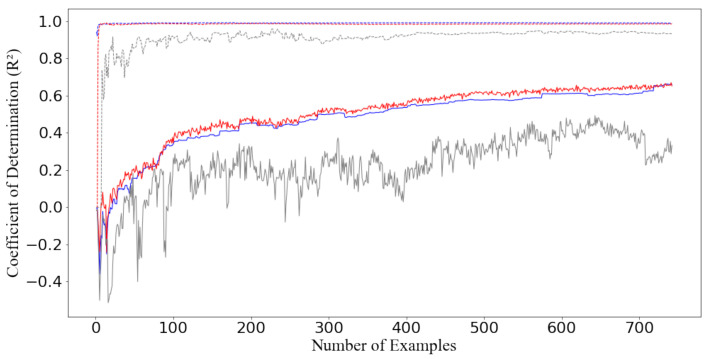
Performances on training (solid lines) and test data (dashed lines) of optimized support vector regressor (blue), random forest regressor (red), and decision tree regressor (grey) evaluated by the coefficient of determination ($R^{2}$).

An illustrative single training procedure of several machine learning models is displayed in Figure 4, which shows the change of $R^{2}$ with respect to the number of instances provided to the algorithms. The test set remained unchanged. The two best models, SVR and RF, are included with a comparison to the DT. Over the first 100 instances imported to the algorithms, SVR and RF display a similar trend: both algorithms quickly reach an $R^{2}$ level of 0.9. Thereafter, the value of $R^{2}$ remains very stable until the end of the training process. The $R^{2}$ for the test set continuously increases with the number of examples, albeit with slight fluctuations. The convergence of $R^{2}$ in the training process seems not to hinder the improvement of test set performance with the inclusion of more instances, which demonstrates that the algorithms continue to recognize the hidden patterns until the end. Therefore, this suggests that the machine learning models might improve further, with additional data.

### 3.3. Performance on Subsets of the Data

**Figure 5:**
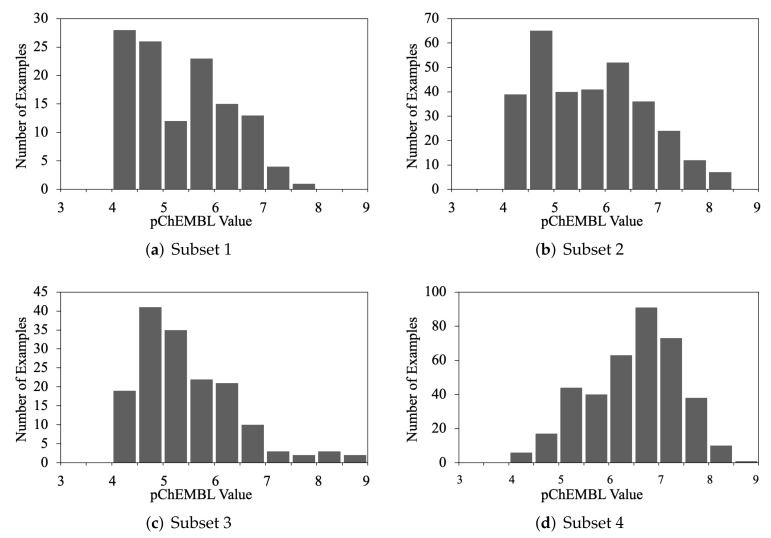
Distribution of the activities in each subset. (**a**) Subset 1: guanidine and thiourea derivatives. (**b**) Subset 2: five or six-membered heterocyclic compounds. (**c**) Subset 3: styrene derivatives. (**d**) Subset 4: tranylcypromine (TCP) derivatives.

In addition to training on the whole dataset, machine learning algorithms were also applied to subsets of the original dataset, in order to explore if the algorithms performed differently on specific structural groups of compounds. Four subsets were selected, and each is based on a representative core structure of known LSD1 inhibitors, giving four distinct (but not necessarily mutually exclusive) subsets [1]. Subset 1 contains guanidine and thiourea derivatives. In the compounds of Subset 2, only selected five or six-membered heterocyclic ring structures are considered. Subset 3 comprises styrene-centered structures. Subset 4 includes all tranylcypromine (TCP) derivatives. As shown in Figure 5, the activity distribution of each subset varies. Subset 4 has the largest fraction of active compounds and the largest number of instances. Generally, all four subsets cover a wide range of activities.

Table 2 shows the performance of machine learning algorithms (the best two and some baseline models) on each individual subset. SVR and RF are the best algorithms, except on Subset 3, in which the ridge regression and SVR perform best. SVR consistently performs well across all the subsets. On the different subsets, the evaluations show noticeable fluctuations in $R^{2}$ and the median RMSE. This may indicate that different subsets of the data manifest structure–activity relationships to varying extents. However, it may be that, in some subsets, the reduced quantity of data compared with the main dataset also impairs the performance of the data-hungry machine learning algorithms. Thus, the performance on the subsets can be less stable than it is on the larger and more diverse full dataset. Therefore, a general machine learning model based on all of the molecules is more advantageous here than several structural-specific models trained on individual subsets.

### 3.4. Virtual Screening

**Figure 6:**
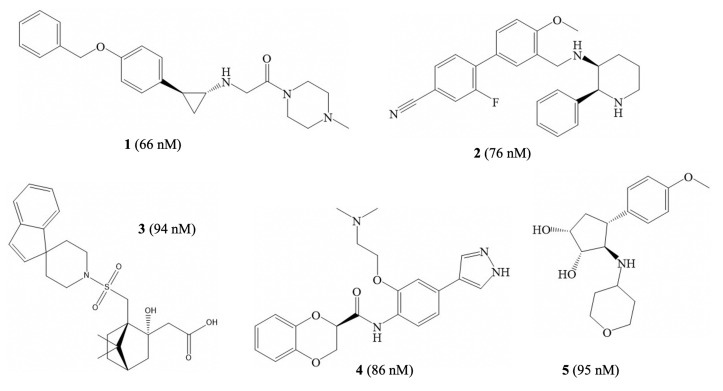
Structures and predicted $IC_{50}$ values (in parentheses) of five molecules produced from the virtual screening: **1** (ZINC000098052700), **2** (ZINC000022449627), **3** (ZINC000038942511), **4** (ZINC000040414461), and **5** (ZINC000072321648).

Virtual screening is our ultimate goal in this study. Molecules were retrieved from the ZINC 15 in-vitro database [43], which contains over 300,000 structures. The model deployed was based on SVR, the best performing algorithm. A threshold of activity was set to 7 (i.e., an effective $IC_{50}$ value of 100 nM), empirically specifying a boundary between ‘active’ and ‘inactive’ molecules in our screen. The virtual screening using the machine learning model resulted in five ‘active’ molecules identified as new structures that were distinct from the 931 molecules in the main dataset. Figure 6 shows the five molecules and their predicted activities. The molecules are quite diverse, with the highest Tanimoto similarity between pairs of molecules being 0.208 (Table S2). The drug-likeness of the predicted molecules was evaluated using Lipinski’s rule of five [46]. Table S1 in the Supplementary Materials shows that the five molecules comply with all the Lipinski criteria.

There are several key structures and functional groups that may be responsible for the activity of the identified molecules, and multiple inhibitory functional groups appear to have been recognized by the algorithm. A five- or six-membered heterocyclic ring, e.g., tetrahydropyran, piperidine, pyrazole, and piperazine, is present in every molecule. Compound **1** belongs to the TCP derivatives by molecular structures. TCP is a major well-known type of irreversible LSD1 inhibitor [47]. The molecule also has a carbonyl piperazine core that possibly enhances the LSD1 inhibition due to a potential hydrogen bond with Asp555 [48]. It also acts as a hydrophobic linker [49]. Compound **2** and Compound **3** both possess the piperidine structure, a functional group that binds to the carboxylate group of Asp555 and the amide oxygen of Asp540 [50]. In addition, a benzonitrile in the terminal part of Compound **2** can act as a selective functional group against LSD1 through the formation of a bridging hydrogen bond with Lys661 [51]. A sulfonamide group, rather than the more common benzenesulfonamide group, is present in Compound **3**, [52], which might indicate that the simpler sulfonamide derivatives should be a future focus. Compound **4** is a heteroaromatic imidazole-based structure. The potency of imidazole against LSD1 has previously been supported by the computational modelling of the binding interactions with the active site of LSD1 [53].

All five molecules were predicted to have high activity. However, due to the upper limit of the activity in the main database, very high values are not likely to be predicted. In Figure 6, Compound **1** is a previously identified LSD1 inhibitor rediscovered by our machine learning model; it is also known as RN1 [47]. In the in vitro assessment of LSD1 inhibition, the $IC_{50}$ value for RN1, as assessed by a horseradish peroxidase (HRP)-coupled assay, is 70 nM [54]. The value is very close to the predicted value of 65.9 nM, illustrating the accuracy of the model. As for the remaining four molecules, our virtual screening indicates their potency against LSD1 for the first time. Two of the compounds are known to have drug-like properties targeting other biological processes, and may be potential cases for drug re-purposing. Compound **2**, known as T-2328, was previously considered an antagonist of tachykinin and the neurokinin-1 receptor [55]. Compound **3**, also known as L-366509, has been considered as a potential antagonist of oxytocin or vasopressin [56]. The other two structures, Compounds **4** and **5**, have not been previously investigated in detail in any other pharmaceutical applications. However, this research suggests that they may be good starting points for the design of new inhibitors of LSD1.

## 4. Conclusions

In this study, a machine learning model was built using data from ChEMBL to enable virtual screening for the discovery of inhibitors of LSD1. The model requires only the structurally based features represented by molecular fingerprints to construct the QSAR between the candidates and the activity of LSD1 inhibition. The final algorithm was selected from several prevailing machine learning algorithms. The best performing algorithm, SVR, reached an average coefficient of determination ($R^{2}$) on the test set of 0.703 on the main dataset, which is a good result from a statistical perspective and gave us the confidence to apply the model in virtual screening. Evaluations on subsets of molecules from the main datasets illustrated that the performance of SVR was more stable than other algorithms, but predictive ability did decline on some of the smaller subsets. The model based on the best performing algorithm was used to discover five molecules with a potential for the inhibition of LSD1 from a large molecular library. We are currently using the model to guide the synthesis of some novel compounds with predicted activity against LSD1.

## Figures and Tables

**Table 1:** Mean performance of each algorithm on predicting the pChEMBL value evaluated by the coefficient of determination ($R^{2}$) and the root mean square error (RMSE). Standard deviations are enclosed in brackets.

Algorithm	Train $R^{2}$	Test $R^{2}$	Train RMSE	Test RMSE
K-Neighbours	0.998 (0.001)	0.662 (0.047)	0.051 (0.010)	0.632 (0.051)
Ridge	0.923 (0.005)	0.471 (0.069)	0.306 (0.011)	0.790 (0.059)
Lasso	0.688 (0.009)	0.597 (0.044)	0.616 (0.010)	0.690 (0.044)
Elastic Net	0.821 (0.006)	0.635 (0.047)	0.466 (0.009)	0.656 (0.047)
Gradient Boosting	0.833 (0.007)	0.631 (0.041)	0.450 (0.010)	0.661 (0.040)
Random Forest	0.984 (0.001)	0.695 (0.035)	0.140 (0.004)	0.600 (0.041)
Adaboost	0.582 (0.017)	0.500 (0.034)	0.713 (0.015)	0.769 (0.035)
Extra Trees	0.998 (0.001)	0.459 (0.092)	0.051 (0.010)	0.798 (0.073)
Decision tree	0.931 (0.009)	0.425 (0.090)	0.288 (0.020)	0.823 (0.066)
SVR	0.989 (0.001)	0.703 (0.035)	0.115 (0.005)	0.592 (0.041)
MLP	0.998 (0.001)	0.544 (0.218)	0.052 (0.010)	0.723 (0.127)

**Table 2:** Mean performance of best two performing and baseline models on predicting the pChEMBL value, evaluated by the coefficient of determination ($R^{2}$) and root mean square error (RMSE).

Dataset	Algorithm	Test $R^{2}$	Test RMSE
Subset 1 ^a^	RF	0.498 (0.172)	0.651 (0.106)
	SVR	0.536 (0.189)	0.623 (0.117)
	DT	0.292 (0.247)	0.772 (0.124)
Subset 2 ^b^	RF	0.760 (0.055)	0.499 (0.057)
	SVR	0.745 (0.055)	0.516 (0.053)
	DT	0.515 (0.133)	0.710 (0.107)
Subset 3 ^c^	Ridge	0.670 (0.141)	0.509 (0.054)
	SVR	0.662 (0.143)	0.516 (0.062)
	DT	0.379 (0.253)	0.701 (0.108)
Subset 4 ^d^	RF	0.458 (0.069)	0.654 (0.053)
	SVR	0.473 (0.081)	0.646 (0.069)
	DT	0.112 (0.171)	0.833 (0.069)

^a^ Subset 1: guanidine and thiourea derivatives. ^b^ Subset 2: six-membered heterocyclic compounds. ^c^ Subset 3: styrene derivatives. ^d^ Subset 4: tranylcypromine (TCP) derivatives.

## Data Availability

The data and scripts for generating the models are available at https://github.com/JiajunZhou96/ML-for-LSD1 (accessed on 9 November 2021).

## Supplementary Materials

The following are available online, Table S1: Predicted pChEMBL values and the Lipinski rule of five properties for the molecules found via virtual screening: 1 (ZINC000098052700), 2 (ZINC000022449627), 3 (ZINC000038942511), 4 (ZINC000040414461), 5 (ZINC000072321648). Table S2: Tanimoto similarities between the molecules identified from virtual screening. The self-similarity of a molecule is, by construction, 1. Table S3: Datasets constructed from different Morgan fingerprints. Table S4: Mean performance of each algorithm on the prediction of pChEMBL values, evaluated by the coefficient of determination (R2) and the root mean square error (RMSE) on Dataset 2. Standard deviations are enclosed in brackets. Table S5: Mean performance of each algorithm on the prediction of pChEMBL values, evaluated by the coefficient of determination (R2) and the root mean square error (RMSE) on Dataset 3. Standard deviations are enclosed in brackets. Table S6: Hyperparameter grids used for optimization. Table S7: Default values of hyperparameters used in the algorithms. Table S8: Best hyperparameters for the machine learning algorithms applied to Dataset 1 (the dataset in the main manuscript). Table S9: Best hyperparameters for the machine learning algorithms applied to Dataset 2. Table S10: Best hyperparameters for the machine learning algorithms applied to Dataset 3. Table S11: Mean performance of each algorithm on the prediction of the pChEMBL values of Subset 1. Standard deviations are enclosed in brackets. Table S12: Mean performance of each algorithm on the prediction of the pChEMBL values of Subset 2. Standard deviations are enclosed in brackets. Table S13: Mean performance of each algorithm on the prediction of the pChEMBL values of Subset 3. Standard deviations are enclosed in brackets. Table S14: Mean performance of each algorithm on the prediction of the pChEMBL values of Subset 4. Standard deviations are enclosed in brackets. Table S15: Best hyperparameters for the machine learning algorithms applied to Subset 1. Table S16: Best hyperparameters for the machine learning algorithms applied to Subset 2. Table S17: Best hyperparameters for the machine learning algorithms applied to Subset 3. Table S18: Best hyperparameters for the machine learning algorithms applied to Subset 4. Figure S1. Train and test performance of optimized support vector regressor (blue), random forest regressor (red), and decision tree regressor (grey) evaluated by the root-mean-square error (RMSE) with dashed lines showing train performances and the solid lines showing test performances, respectively. Figure S2. The neural network architecture applied to a dataset with 512 inputs. Figure S3. Schematic of back propagation in the multi-layer perceptron applied to a dataset with 512 inputs. Figure S4. Core structures for Subsets 1, 2, 3 and 4, respectively: (a) guanidine and thiourea derivatives; (b) molecules containing selected five or six-membered heterocycles; (c) styrene derivatives. (d) tranylcypromine (TCP) derivatives.

## Sample Availability

Samples of the compounds are not available from the authors.

## Abbreviations

The following abbreviations are used in this manuscript:

DT	Decision tree regressor
HRP	Horseradish peroxidase
LSD1	Lysine-specific histone demethylase 1
MAO	Monoamine oxidases
MLP	Multi-layer perceptron
QSAR	Quantitative structure-activity relationship
RBF	Radial basis function
RF	Random forest regressor
RMSE	Root mean square error
SMILES	Simplified Molecular-Input Line-Entry System
SVR	Support vector regressor
t-SNE	t-distributed Stochastic Neighbour Embedding
TCP	Tranylcypromine
