# Supplementary material for: Machine-Learning-Enabled Virtual Screening for Inhibitors of Lysine-Specific Histone Demethylase 1
Source: Molecules. 2021 Dec 10;26(24):7492. doi: 10.3390/molecules26247492 (PMC8707381; doi:10.3390/molecules26247492)
Supplement: Supplementary file 1 [file molecules-26-07492-s001.zip › molecules-1460158-supplementary - XML done.pdf]

molecules

Article

Machine Learning Enabled Virtual Screening for Inhibitors of Lysine-Specific Histone Demethylase 1

Jiajun Zhou [1], Shiying Wu [1], Boon Giin Lee [2], Tianwei Chen [1], Ziqi He [1], Yukun Lei [1], Bencan Tang [1,*] and Jonathan D. Hirst [3,*]

1 Key Laboratory for Carbonaceous Waste Processing and Process Intensification Research of Zhejiang Province, University of Nottingham Ningbo China, 199 Taikang East Road, Ningbo, 315100, China
2 School of Computer Science, University of Nottingham Ningbo China, 199 Taikang East Road, Ningbo, 315100, China
3 School of Chemistry, University of Nottingham, University Park, Nottingham, NG7 2RD, United Kingdom
* Correspondence: bencan.tang@nottingham.edu.cn (B.T.); jonathan.hirst@nottingham.ac.uk (J.H.)

Citation: Zhou, J.; Wu, S.; Lee, B.; Chen, T.; He, T; Lei, Y.; Tang, B.; Hirst, J.D. Machine Learning Enabled Virtual Screening for Inhibitors of Lysine-Specific Histone Demethylase 1. *Molecules* **2021**, *26*, 7492. https://doi.org/10.3390/molecules26247492

Received: 27 October 2021
Accepted: 6 December 2021
Published: 10 December 2021

Publisher's Note: MDPI stays neutral with regard to jurisdictional claims in published maps and institutional affiliations.

Copyright: © 2021 by the authors. Licensee MDPI, Basel, Switzerland. This article is an open access article distributed under the terms and conditions of the Creative Commons Attribution (CC BY) license (https://creativecommons.org/licenses/by/4.0/).

1. Molecular Dataset

The SMILES representations of molecules in all the datasets are included in separate Excel files.

2. Lipinski's Rule of Five

Lipinski's rule of five was used to evaluate the druglikeness of the five molecules identified by screening the ZINC database. This rule of thumb comprises the following criteria: (1) the molecular mass of the molecule is less than 500 DA; (2) the calculated partition coefficient (*log P*) is less than 5; (3) no more than 5 hydrogen bond donors; (4) no more than 10 hydrogen bond acceptors.

The pChEMBL values for the inhibition activity of LSD1 were predicted by our model. The octanol-water partition coefficient (*log P*) was taken directly from ZINC 15 [1].

Compound	pChEMBL Value	Mass <500	*log P* <5	H-bond Donor<5	H-bond Acceptor<10
**1**	7.18	379.50	2.49	1	4
**2**	7.12	415.51	4.96	2	3
**3**	7.03	459.61	3.41	2	4
**4**	7.07	408.46	2.80	2	6
**5**	7.02	307.39	1.04	3	2

Table S1: Predicted pChEMBL values and the Lipinski rule of five properties for the molecules found via virtual screening: **1** (ZINC000098052700), **2** (ZINC000022449627), **3** (ZINC000038942511), **4** (ZINC000040414461), **5** (ZINC000072321648).

3. Similarity Between Molecules

The Tanimoto coefficients were calculated from the Morgan fingerprints. Here, similarities between every pair of the five structures are shown in Table S2.

Structure A	Structure B	Tanimoto Similarity
**1**	**1**	1.000
**1**	**2**	0.201
**1**	**3**	0.158
**1**	**4**	0.170
**1**	**5**	0.208
**2**	**2**	1.000
**2**	**3**	0.107
**2**	**4**	0.093
**2**	**5**	0.131
**3**	**3**	1.000
**3**	**4**	0.116
**3**	**5**	0.104
**4**	**4**	1.000
**4**	**5**	0.178
**5**	**5**	1.000

Table S2: Tanimoto similarities between the molecules identified from virtual screening. The self-similarity of a molecule is, by construction, 1.

4. Evaluation of Algorithms

The predictive accuracy was evaluated by two metrics: the coefficient of determination (R^2) and root-mean-square error (RMSE). RMSE is defined in Equation (S1). The error in prediction is represented by the difference between predicted value for observation i ($\hat{y}_i$) and actual value (y_i). The best value of RMSE is zero, indicating the minimal limit of possible error.

$$RMSE = \sqrt{\sum_{i=1}^{n} \frac{(\hat{y}_i - y_i)^2}{n}} \tag{S1}$$

R^2 is defined as the ratio of the sum of squared residual errors (SSR) and the total sum of squares (SST). The total variance in system can be traced back to two sources: the explained variance by regression and remaining residue errors. Therefore we could also express the definition of R^2 as the first equation in Equation (S2). $\bar{y}$ represents the average of all actual values.

$$\begin{aligned} R^2 &= 1 - \frac{SSE}{SST} \\ SSE &= \sum_{i=1}^{n} (y_i - \hat{y}_i)^2 \\ SST &= \sum_{i=1}^{n} (y_i - \bar{y})^2 \end{aligned} \tag{S2}$$

For the RMSE and R^2 of each run on a particular algorithm, the average and population standard deviation were calculated to help characterise the overall performance.

5. Performance of Algorithms on Main Dataset

Figure 1 shows the RMSE performance on the main dataset for the support vector regressor (SVR), the random forest regressor (RF) and the decision tree regressor (DT).

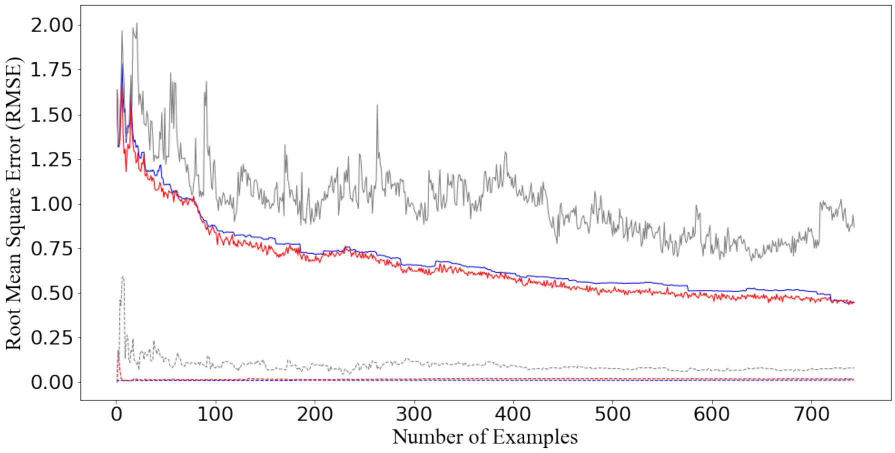

Figure 1. Train and test performance of optimized support vector regressor (blue), random forest regressor (red), and decision tree regressor (grey) evaluated by the root-mean-square error (RMSE) with dashed lines showing train performances and the solid lines showing test performances, respectively.

6. Additional Datasets

Three datasets encoded by different radius and length of bit vector are shown in Table S3. They were used to compare the impact of radius and the length of bit vector on the construction of feature space, as the performance of machine learning algorithms can be influenced by the representation of data. Dataset 1 is the main dataset assessed in the manuscript. Datasets 2 and 3 are additional datasets, on which the performance of the algorithms was assessed, as described in the following section.

Dataset	Radius	Length of Bit Vector	Dataset Dimensions
Dataset 1	3	512	(931, 512)
Dataset 2	2	512	(931, 512)
Dataset 3	3	1024	(931, 1024)

Table S3: Datasets constructed from different Morgan fingerprints.

7. Performances of Algorithms on Additional Datasets

The algorithm performances on the additional datasets, Datasets 2 and 3, are shown in Tables S4 and S5. The best two algorithms on the test set are SVR and RF for both Datasets 2 and 3. Comparing the predictions on Datasets 1 and 2, the larger radius seems to improve the accuracy of the major machine learning algorithms. This may be because the larger radius encodes more information in the bit vector about the neighbouring atoms and environments. In the comparison of Dataset 3 to Dataset 1, the predictive accuracies are very similar, even though the number of bits in the bit vectors is doubled.

**Algorithm**	**Train R^2**	**Test R^2**	**Train RMSE**	**Test RMSE**
K-Neighbours	0.997 (0.001)	0.648 (0.052)	0.062 (0.009)	0.644 (0.055)
Ridge	0.889 (0.005)	0.541 (0.066)	0.368 (0.008)	0.736 (0.053)
Lasso	0.646 (0.009)	0.576 (0.041)	0.656 (0.009)	0.708 (0.040)
Elastic Net	0.745 (0.008)	0.625 (0.046)	0.557 (0.010)	0.666 (0.045)
Gradient Boosting	0.796 (0.006)	0.628 (0.039)	0.498 (0.009)	0.663 (0.039)
Random Forest	0.975 (0.002)	0.685 (0.037)	0.174 (0.007)	0.610 (0.043)
Adaboost	0.545 (0.027)	0.474 (0.036)	0.744 (0.023)	0.789 (0.032)
Extra Trees	0.997 (0.001)	0.454 (0.084)	0.062 (0.009)	0.802 (0.066)
Decision tree	0.988 (0.003)	0.412 (0.092)	0.121 (0.014)	0.833 (0.070)
SVR	0.977 (0.001)	0.696 (0.037)	0.166 (0.006)	0.599 (0.042)
MLP	0.997 (0.001)	0.513 (0.080)	0.064 (0.009)	0.757 (0.059)

Table S4: Mean performance of each algorithm on the prediction of pChEMBL values, evaluated by the coefficient of determination (R^2) and the root mean square error (RMSE) on Dataset 2. Standard deviations are enclosed in brackets.

**Algorithm**	**Train R^2**	**Test R^2**	**Train RMSE**	**Test RMSE**
K-Neighbours	0.998 (0.001)	0.664 (0.046)	0.051 (0.010)	0.630 (0.051)
Ridge	0.969 (0.002)	0.506 (0.057)	0.194 (0.006)	0.764 (0.044)
Lasso	0.921 (0.003)	0.576 (0.045)	0.310 (0.007)	0.708 (0.043)
Elastic Net	0.836 (0.005)	0.645 (0.041)	0.447 (0.007)	0.647 (0.041)
Gradient Boosting	0.809 (0.007)	0.619 (0.035)	0.482 (0.009)	0.671 (0.036)
Random Forest	0.983 (0.001)	0.693 (0.037)	0.145 (0.004)	0.602 (0.042)
Adaboost	0.532 (0.011)	0.456 (0.036)	0.754 (0.011)	0.803 (0.033)
Extra Trees	0.998 (0.001)	0.467 (0.073)	0.051 (0.010)	0.793 (0.053)
Decision tree	0.977 (0.003)	0.423 (0.079)	0.166 (0.013)	0.825 (0.053)
SVR	0.989 (0.001)	0.703 (0.033)	0.115 (0.005)	0.593 (0.038)
MLP	0.998 (0.001)	0.552 (0.087)	0.052 (0.010)	0.726 (0.070)

Table S5: Mean performance of each algorithm on the prediction of pChEMBL values, evaluated by the coefficient of determination (R^2) and the root mean square error (RMSE) on Dataset 3. Standard deviations are enclosed in brackets.

8. Hyperparameter Grid

For each algorithm, several key hyperparameters were chosen to form the hyperparameter grid for optimization (shown in Table S6). Other hyperparameters were fixed at their default settings (shown in Table S7). Five-fold cross validation process on the training set was used in the optimization process. The optimized hyperparameters were subsequently used to construct the algorithms for further training and testing.

Algorithm	Hyperparamter Grid
K-Neighbours	'n_neighbors': {2,3,4,5,6,7,8,9,10 },<br>'weights': {'uniform', 'distance'},<br>'algorithm': {'auto', 'ball_tree', 'kd_tree', 'brute'},<br>'leaf_size': {20, 30, 40}, 'p': {1,2}
Ridge	'alpha': {0.001,0.01,0.1,1,10}
Lasso	'alpha': {0.001,0.01,0.1,1,10},<br>'selection':{'cyclic', 'random'}
Elastic Net	'alpha': {0.001,0.01,0.1,1,10},<br>'l1_ratio':{0.2, 0.3, 0.4, 0.5, 0.6, 0.7, 0.8}
Gradient Boosting	'loss': {'ls', 'lad', 'huber', 'quantile'},<br>'learning_rate': {0.001,0.01,0.1,1 },<br>'criterion':{'mse', 'friedman_mse'},<br>'min_samples_split': {2,3,4,5,6,7,8,9}
Ada Boost	'loss': {'linear', 'square', 'exponential'},<br>'learning_rate': {0.001,0.01,0.1,1 }
Extra Trees	'bootstrap': {True, False},<br>'min_samples_split': {2,3,4,5,6,7,8,9}
Random Forest	'bootstrap': {True, False},<br>'max_features':{'auto','log2','sqrt'}<br>'min_samples_split': {2,3,4,5,6,7,8,9}
Decision Tree	'criterion': {'mse', 'friedman_mse'}<br>'min_samples_split': {2,3,4,5,6,7,8,9}
SVR	'kernel': {'rbf', 'linear', 'poly', 'sigmoid'},<br>'gamma': {'scale', 'auto'},<br>'C': {0.001, 0.01, 0.1, 1, 10, 20, 50, 100}

Table S6: Hyperparameter grids used for optimization

Algorithm	Default Parameters
K-Neighbours	metric = 'minkowski', metric_params = None, n_jobs = None
Ridge	fit_intercept = True, normalize = False, copy_X = True, max_iter = 100000, tol = 1e-3, solver = 'auto', random_state = None
Lasso	fit_intercept = True, normalize = False, precompute = False, copy_X = True, max_iter = 100000, tol = 1e-4, warm_start = False, positive = False, random_state = None
Elastic Net	fit_intercept = True, normalize = False, precompute = False, copy_X = True, max_iter = 100000, tol = 1e-4, warm_start = False, positive = False, random_state = None, selection = 'cyclic'
Gradient Boosting	n_estimators = 100, subsample = 1.0, min_samples_leaf = 1, min_weight_fraction_leaf = 0.0, max_depth = 3 min_impurity_decrease = 0.0, min_impurity_split = None, init = None, random_state = None, max_features = None, verbose = 0, max_leaf_nodes = None, warm_start = False, validation_fraction = 0.1, n_iter_no_change = None, tol = 1e-4, ccp_alpha = 0.0
Ada Boost	base_estimator = None, n_estimators = 50, random_state = None
Extra Trees	n_estimators = 100, criterion = 'mse', max_depth = None, min_samples_leaf = 1, min_weight_fraction_leaf = 0.0, max_features = "auto", max_leaf_nodes = None, min_impurity_decrease = 0.0, min_impurity_split = None, oob_score = False, n_jobs = None, random_state = None, verbose = 0, warm_start = False, ccp_alpha = 0.0, max_samples = None
Random Forest	n_estimators = 100, criterion = 'mse', max_depth = None, min_samples_leaf = 1, min_weight_fraction_leaf = 0.0, max_features = "auto", max_leaf_nodes = None, min_impurity_decrease = 0.0, min_impurity_split = None, oob_score = False, n_jobs = None, random_state = None, verbose = 0, warm_start = False, ccp_alpha = 0.0, max_samples = None
Decision Tree	splitter = 'best', max_depth = None, min_samples_leaf = 1, min_weight_fraction_leaf = 0.0, max_features = None, random_state = None, max_leaf_nodes = None, min_impurity_decrease = 0.0, min_impurity_split = 0, ccp_alpha =0.0
SVR	degree = 3, coef0 = 0.0, tol = 1e-3, epsilon = 0.1, shrinking = True, cache_size = 200, verbose = False, max_iter = -1

Table S7: Default values of hyperparameters used in the algorithms

9. Best Hyperparameters for the Main and Additional Datasets

For all three datasets, due to the difference in the fingerprints used, the optimal hyperparameters of the machine learning algorithms are also different. The optimized hyperparameters for the main implementation are shown in Table S8, while those for Datasets 1 and 2 are shown in Tables S9 and S10, respectively.

Algorithm	Best Hyperparameters
K-Neighbours	n_neighbors = 6, weights = 'distance', algorithm = 'ball_tree', leaf_size = 20, p = 1
Ridge	alpha = 1.0
Lasso	alpha = 0.01, selection = 'cyclic'
Elastic Net	alpha = 0.01, l1_ratio = 0.2
Gradient Boosting	loss = 'huber', learning_rate = 0.1, criterion = 'friedman_mse', min_samples_split = 3
Random Forest	min_samples_split = 4, max_features = 'sqrt', bootstrap = False
Ada Boost	learning_rate = 1, loss = 'linear'
Extra Trees	min_samples_split = 2, bootstrap = False
Decision Trees	criterion='friedman_mse', min_samples_split = 9
SVR	kernel = 'rbf', gamma = 'scale', C = 10

Table S8: Best hyperparameters for the machine learning algorithms applied to Dataset 1 (the dataset in the main manuscript).

Algorithm	Best Hyperparameters
K-Neighbours	n_neighbors = 6, weights = 'distance', algorithm = 'brute', leaf_size = 20, p = 1
Ridge	alpha = 1.0
Lasso	alpha = 0.01, selection = 'cyclic'
Elastic Net	alpha = 0.01, l1_ratio = 0.3
Gradient Boosting	loss = 'ls', learning_rate = 0.1, criterion = 'mse', min_samples_split = 3
Random Forest	min_samples_split = 3, max_features = 'log2', bootstrap = False
Ada Boostr	learning_rate = 1, loss = 'square'
Extra Trees	min_samples_split = 2, bootstrap = False
Decision Trees	criterion='mse', min_samples_split = 7
SVR	kernel = 'rbf', gamma = 'scale', C = 10

Table S9: Best hyperparameters for the machine learning algorithms applied to Dataset 2.

Algorithms	Best Hyperparameters
K-Neighbours	n_neighbors = 7, weights = 'distance', algorithm = 'brute', leaf_size = 20, p = 1
Ridge	alpha = 1.0
Lasso	alpha = 0.01, selection = 'cyclic'
Elastic Net	alpha = 0.01, l1_ratio = 0.2
Gradient Boosting	loss = 'huber', learning_rate = 0.1, criterion = 'mse', min_samples_split = 3
Random Forest	min_samples_split = 4, max_features = 'sqrt', bootstrap = False
Ada Boost	learning_rate = 0.1, loss = 'square'
Extra Trees	min_samples_split = 2, bootstrap = False
Decision Trees	criterion='mse', min_samples_split = 5
SVR	kernel = 'rbf', gamma = 'scale', C = 10

Table S10: Best hyperparameters for the machine learning algorithms applied to Dataset 3.

10. Neural Network Architecture

In this study, a neural network method was also applied to the main and additional datasets. A pre-determined architecture with a unified hyperparameter set was utilized. As shown in Figure 2, the type of neural network employed is a multi-layer perceptron (MLP) with two hidden layers. Each hidden layer integrates a linear module and a subsequent batch normalization. The Leaky Rectified Linear Unit (Leaky ReLU) was utilized as the activation function in both hidden layers. The size of layers continuously decreases with the depth of neural network, from the number of the features in the input layer to the single continuous variable in the output layer [1]. A 1024-unit input layer was required for Dataset 3; both Datasets 1 and 2 had 512 units in the input layer for the MLP. The MSE loss function was chosen to monitor the loss during training of the neural network. The weights were optimized with the Adam optimization algorithm, which combines the advantages of Momentum and RMSProp algorithms [2]. The training of the MLP relies on back propagation, as shown schematically in Figure 3. The evaluation metrics are the same, for the convenience of comparison.

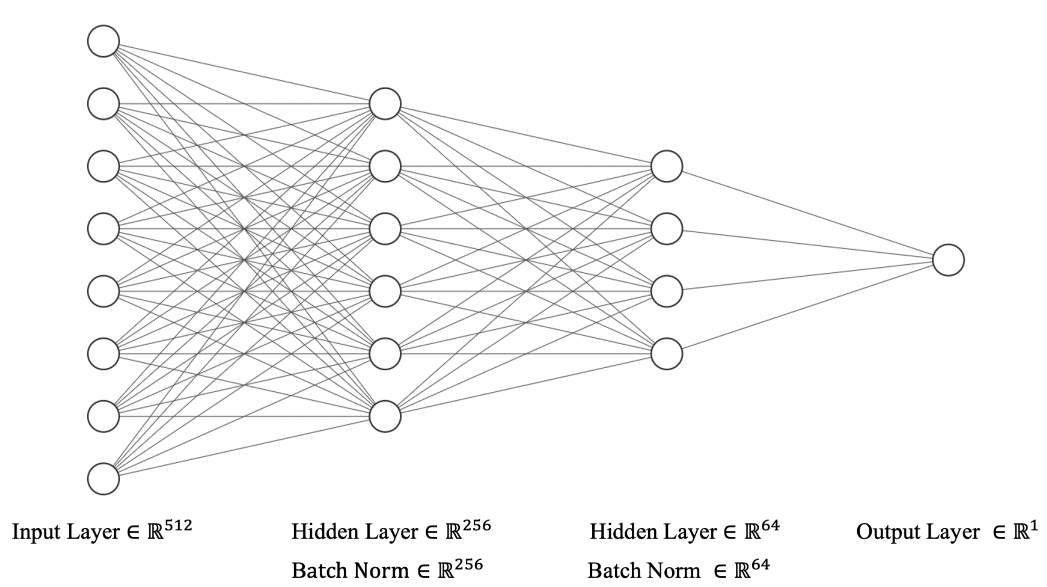

Figure 2. The neural network architecture applied to a dataset with 512 inputs.

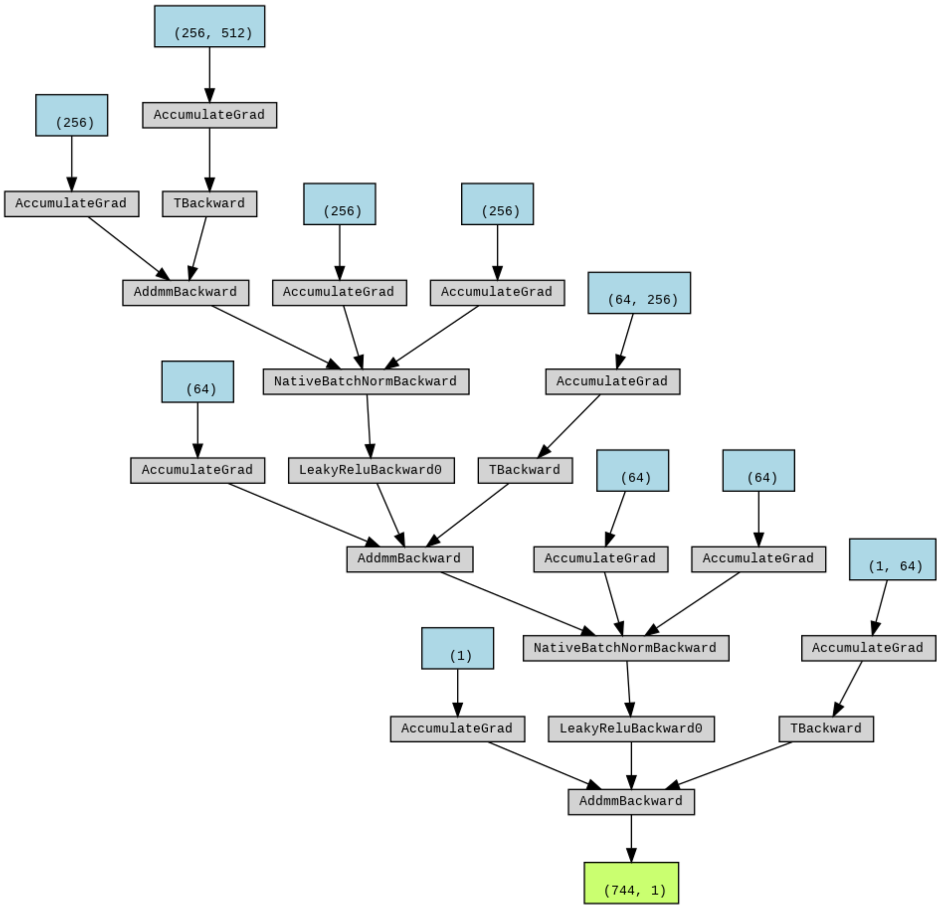

Figure 3. Schematic of back propagation in the multi-layer perceptron applied to a dataset with 512 inputs.

11. Subsets of the main dataset

Figure 4 shows the core structures in four manually divided subsets. In Figure 4 (c), the substituent group R_3 can take the *para*, *meta* or sometimes *ortho* position with respect to the ethylene substituent. It is also possible to have extra two substituent groups besides the ethylene group. In Figure 4 (d), one of the three substituent groups is directly connected with a benzene ring. Although the main manuscript only lists the best two performing models and the baseline models, the performances of all the algorithms are shown in Tables S11, S12, S13 and S14.

The default hyperparameters and hyperparameter grids for tuning the algorithms applied to the data subsets are in accordance with Table S6 and S7. Due to different distributions in subsets, the set of best hyperparameters also changes for the best fitting under the similar training and tuning procedures compared with the complete datasets. The best hyperparameters are shown in Tables S15, S16, S17 and S18.

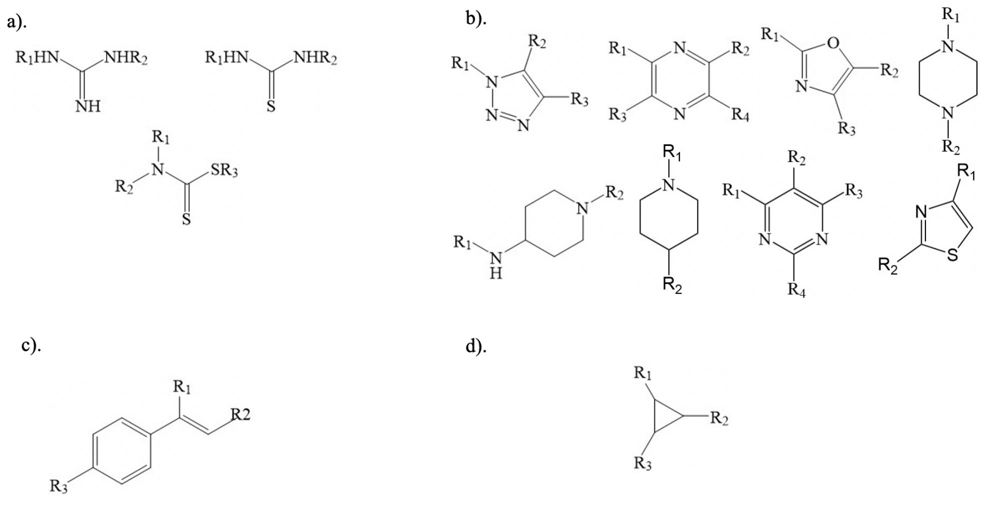

Figure 4. Core structures for Subsets 1, 2, 3 and 4, respectively: (a) guanidine and thiourea derivatives; (b) molecules containing selected five or six-membered heterocycles; (c) styrene derivatives. (d) tranylcypromine (TCP) derivatives.

Algorithm Types	Train R^2	Test R^2	Train RMSE	Test RMSE
K-Neighbours	0.993 (0.003)	0.470 (0.167)	0.077 (0.022)	0.670 (0.106)
Ridge	0.978 (0.004)	0.492 (0.189)	0.136 (0.015)	0.654 (0.114)
Lasso	0.863 (0.013)	0.428 (0.181)	0.344 (0.016)	0.697 (0.113)
Elastic Net	0.946 (0.007)	0.458 (0.203)	0.216 (0.013)	0.675 (0.118)
Gradient Boosting	0.907 (0.047)	0.377 (0.215)	0.275 (0.071)	0.725 (0.131)
Random Forest	0.894 (0.013)	0.498 (0.172)	0.303 (0.018)	0.651 (0.106)
Adaboost	0.778 (0.032)	0.419 (0.198)	0.438 (0.031)	0.700 (0.124)
Extra Trees	0.993 (0.003)	0.361 (0.267)	0.077 (0.022)	0.727 (0.136)
Decision tree	0.896 (0.029)	0.292 (0.247)	0.298 (0.043)	0.772 (0.125)
SVR	0.898 (0.016)	0.536 (0.189)	0.396 (0.025)	0.623 (0.117)

Table S11: Mean performance of each algorithm on the prediction of the pChEMBL values of Subset 1. Standard deviations are enclosed in brackets.

Algorithm	Train R^2	Test R^2	Train RMSE	Test RMSE
K-Neighbours	0.800 (0.016)	0.697 (0.101)	0.477 (0.019)	0.556 (0.084)
Ridge	0.976 (0.003)	0.669 (0.075)	0.165 (0.008)	0.597 (0.044)
Lasso	0.829 (0.008)	0.679 (0.073)	0.440 (0.010)	0.578 (0.066)
Elastic Net	0.894 (0.005)	0.694 (0.069)	0.346 (0.008)	0.564 (0.059)
Gradient Boosting	0.938 (0.007)	0.700 (0.081)	0.264 (0.015)	0.557 (0.072)
Random Forest	0.973 (0.002)	0.760 (0.055)	0.173 (0.007)	0.499 (0.057)
Adaboost	0.785 (0.013)	0.661 (0.069)	0.493 (0.014)	0.594 (0.054)
Extra Trees	0.997 (0.002)	0.561 (0.142)	0.052 (0.024)	0.672 (0.113)
Decision tree	0.993 (0.002)	0.515 (0.133)	0.085 (0.016)	0.710 (0.107)
SVR	0.987 (0.002)	0.745 (0.055)	0.122 (0.011)	0.516 (0.053)

Table S12: Mean performance of each algorithm on the prediction of the pChEMBL values of Subset 2. Standard deviations are enclosed in brackets.

**Algorithm**	**Train R^2**	**Test R^2**	**Train RMSE**	**Test RMSE**
K-Neighbours	1.000 (0.000)	0.562 (0.197)	0.000 (0.000)	0.586 (0.090)
Ridge	0.994 (0.001)	0.670 (0.141)	0.073 (0.005)	0.509 (0.054)
Lasso	0.899 (0.011)	0.610 (0.176)	0.298 (0.011)	0.551 (0.075)
Elastic Net	0.963 (0.003)	0.632 (0.162)	0.181 (0.0060)	0.537 (0.067)
Gradient Boosting	0.972 (0.003)	0.606 (0.177)	0.158 (0.009)	0.554 (0.059)
Random Forest	0.946 (0.007)	0.594 (0.179)	0.218 (0.010)	0.564 (0.072)
Adaboost	0.753 (0.026)	0.598 (0.155)	0.466 (0.017)	0.565 (0.079)
Extra Trees	1.000 (0.000)	0.421 (0.228)	0.000 (0.000)	0.679 (0.099)
Decision tree	0.984 (0.006)	0.379 (0.253)	0.117 (0.022)	0.701 (0.108)
SVR	0.989 (0.001)	0.662 (0.143)	0.099 (0.003)	0.516 (0.062)

Table S13: Mean performance of each algorithm on the prediction of the pChEMBL values of Subset 3. Standard deviations are enclosed in brackets.

**Algorithm**	**Train R^2**	**Test R^2**	**Train RMSE**	**Test RMSE**
K-Neighbours	0.676 (0.020)	0.368 (0.089)	0.516 (0.013)	0.706 (0.054)
Ridge	0.963 (0.003)	0.157 (0.171)	0.175 (0.009)	0.815 (0.097)
Lasso	0.659 (0.014)	0.354 (0.088)	0.530 (0.010)	0.715 (0.064)
Elastic Net	0.816 (0.008)	0.362 (0.096)	0.389 (0.010)	0.710 (0.065)
Gradient Boosting	0.871 (0.010)	0.408 (0.057)	0.326 (0.014)	0.685 (0.050)
Random Forest	0.900 (0.005)	0.458 (0.069)	0.287 (0.009)	0.654 (0.053)
Adaboost	0.505 (0.090)	0.328 (0.066)	0.638 (0.020)	0.730 (0.061)
Extra Trees	0.998 (0.001)	0.194 (0.147)	0.041 (0.015)	0.795 (0.061)
Decision tree	0.913 (0.018)	0.112 (0.171)	0.266 (0.028)	0.833 (0.069)
SVR	0.987 (0.001)	0.473 (0.081)	0.104 (0.006)	0.646 (0.069)

Table S14: Mean performance of each algorithm on the prediction of the pChEMBL values of Subset 4. Standard deviations are enclosed in brackets.

Algorithm	Best Hyperparameters
K-Neighbours	n_neighbors = 8, weights = 'distance', algorithm = 'brute', leaf_size = 20, p = 1
Ridge	alpha = 1.0
Lasso	alpha = 0.01, selection = 'random'
Elastic Net	alpha = 0.01, l1_ratio = 0.3
Gradient Boosting	loss = 'lad', learning_rate = 1, criterion = 'mse', min_samples_split = 2
Random Forest	min_samples_split = 6, max_features = 'log2', bootstrap = False
Ada Boost	learning_rate = 0.001, loss = 'linear'
Extra Trees	min_samples_split = 2, bootstrap = False
Decision Trees	criterion='friedman_mse', min_samples_split = 9
SVR	kernel = 'poly', gamma = 'scale', C = 10

Table S15: Best hyperparameters for the machine learning algorithms applied to Subset 1.

Algorithm	Best Hyperparameters
K-Neighbours	n_neighbors = 5, weights = 'uniform', algorithm = 'ball_tree', leaf_size = 30, p = 1
Ridge	alpha = 1.0
Lasso	alpha = 0.01, selection = 'random'
Elastic Net	alpha = 0.01, l1_ratio = 0.4
Gradient Boosting	loss = 'ls', learning_rate = 0.1, criterion = 'mse', min_samples_split = 5
Random Forest	min_samples_split = 5, max_features = 'sqrt', bootstrap = False
Ada Boostr	learning_rate = 1, loss = 'exponential'
Extra Trees	min_samples_split = 2, bootstrap = False
Decision Trees	criterion='friedman_mse', min_samples_split = 3
SVR	kernel = 'rbf', gamma = 'scale', C = 10

Table S16: Best hyperparameters for the machine learning algorithms applied to Subset 2.

Algorithm	Best Hyperparameters
K-Neighbours	n_neighbors = 6, weights = 'distance', algorithm = 'ball_tree', leaf_size = 20, p = 1
Ridge	alpha = 1.0
Lasso	alpha = 0.01, selection = 'random'
Elastic Net	alpha = 0.01, l1_ratio = 0.3
Gradient Boosting	loss = 'ls', learning_rate = 0.1, criterion = 'mse', min_samples_split = 5
Random Forest	min_samples_split = 3, max_features = 'auto', bootstrap = True
Ada Boost	learning_rate = 0.001, loss = 'exponential'
Extra Treesr	min_samples_split = 2, bootstrap = False
Decision Trees	criterion='mse', min_samples_split = 4
SVR	kernel = 'rbf', gamma = 'auto', C = 50

Table S17: Best hyperparameters for the machine learning algorithms applied to Subset 3.

Algorithm	Best Hyperparameters
K-Neighbours	n_neighbors = 4, weights = 'uniform', algorithm = 'auto', leaf_size = 20, p = 1
Ridge	alpha = 1.0
Lasso	alpha = 0.01, selection = 'random'
Elastic Net	alpha = 0.01, l1_ratio = 0.3
Gradient Boosting	loss = 'ls', learning_rate = 0.1, criterion = 'mse', min_samples_split = 5
Random Forest	min_samples_split = 5, max_features = 'auto', bootstrap = True
Ada Boost	learning_rate = 0.c1, loss = 'exponential'
Extra Trees	min_samples_split = 2, bootstrap = False
Decision Trees	criterion='friedman_mse', min_samples_split = 8
SVR	kernel = 'rbf', gamma = 'scale', C = 20

Table S18: Best hyperparameters for the machine learning algorithms applied to Subset 4.

References

1. Sterling, T.; Irwin, J.J. ZINC 15–ligand discovery for everyone. *J. Chem. Inf. Model.* **2015**, *55*, 2324–2337.
2. Kingma, D.P.; Ba, J. Adam: A Method for Stochastic Optimizatio. 3rd International Conference on Learning Representations, *ICLR* 2015, San Diego, CA, USA, May 7–9, 2015, Conference Track Proceedings, 2015.
